# Taking common ground into account: Specifying the role of the mentalizing network in communicative language production

**DOI:** 10.1371/journal.pone.0202943

**Published:** 2018-10-11

**Authors:** Flora Vanlangendonck, Roel M. Willems, Peter Hagoort

**Affiliations:** 1 Donders Institute for Brain, Cognition and Behaviour, Radboud University, Nijmegen, The Netherlands; 2 International Max Planck Research School for Language Sciences, Nijmegen, The Netherlands; 3 Max Planck Institute for Psycholinguistics, Nijmegen, The Netherlands; 4 Centre for Language Studies, Radboud University, Nijmegen, The Netherlands; Bangor University, UNITED KINGDOM

## Abstract

Several studies have shown that communicative language production as compared to non-communicative language production recruits parts of the mentalizing or theory of mind network, yet the exact role of this network in communication remains underspecified. In this study, we therefore aimed to test under what conditions the mentalizing network contributes to communicative language production. We were especially interested in distinguishing between situations in which speakers have to consider which information they do or do not share with their addressee (common vs. privileged ground information). We therefore manipulated whether speakers had to distinguish between common and privileged ground in order to communicate efficiently with the listener, in addition to comparing language production in a communicative and a non-communicative context. Participants performed a referential communicative game in the MRI-scanner as well as a similar, non-communicative task. We found that the medial prefrontal cortex, a core region of the mentalizing network, is especially sensitive to communicative contexts in which speakers have to take their addressee’s needs into account in order to communicate efficiently. In addition, we found neural differences between the communicative and the non-communicative settings before speakers started to plan their utterances, suggesting that they continuously update common ground in a communicative context.

## Introduction

Recent years have seen an increased interest in the study of the neural mechanisms supporting the social and communicative aspects of language production. A number of studies have shown that planning a communicative action as compared to a non-communicative action recruits parts of the mentalizing or theory of mind network, suggesting that people mentalize about their interlocutor’s intentions and beliefs when planning a communicative action. For example, language production in a communicative task as compared to a non-communicative task was found to recruit parts of the mentalizing network: the temporoparietal junction / superior temporal sulcus (TPJ/pSTS), and the medial prefrontal cortex (mPFC; [1,2]). Similarly, in a non-linguistic context, the pSTS is more activated when planning a communicative action compared to a non-communicative action [3] and during communicative pointing compared to non-communicative pointing [4].

But what sets apart communicative from non-communicative language production? In a communicative context, speakers typically have to take into account what information they share with their addressee (common ground) and what information they do not share (privileged ground). For example, when explaining the results of your latest experiment to a colleague, you should take into account how much this person knows about your research topic and whether you have talked about it before. Such adjustments require that you monitor common ground to create a model of what knowledge and beliefs you have in common with your interlocutor [5–7]. Some information for the model may be available from the start of the interaction, while other information only becomes available as the interaction unfolds [8]. For example, while explaining the results of your experiment to your colleague, you may discover that they know less about the topic than you anticipated, requiring you to adjust your language use accordingly. Here we extend previous studies on the neural basis of communicative language production by explicitly manipulating common and privileged ground in a language production task.

We examined the neural mechanisms that allow speakers to adapt their language use to moment-to-moment changes in common ground by manipulating which information they do or do not share with their addressee, in addition to comparing language production in a communicative and a non-communicative context. We used a well-established paradigm that allows for tight control over the linguistic utterances that are produced. Speakers in the MRI scanner either described objects to a listener outside the scanner (*communicative blocks*) or for themselves (*non-communicative blocks*), which allowed us to tap into the process of building a model of your addressee. In addition, and most importantly, within the communicative blocks, we manipulated whether the speaker had to distinguish between common and privileged ground in order to communicate efficiently with the listener (*privileged ground vs*. *control conditions*). In the privileged ground condition, speakers saw additional competitor objects that were occluded from the addressee’s point of view. They had to take into account that the addressee could not see these privileged-ground objects in order to communicate clearly with the addressee. In the control conditions, all relevant objects were mutually visible.

Previous psycholinguistic research has shown that speakers are generally able to distinguish between common and privileged ground information in their utterances, but that they cannot completely ignore privileged ground information (e.g., [9–13]). For example, in a previous eye-tracking study using the same paradigm as the communicative blocks in the current study [10], we found that speakers generally take their addressee’s perspective into account in privileged ground trials, but sometimes produce descriptions from their own perspective. We found no evidence that taking your addressee’s perspective into account results in longer planning durations. In the current study, we expected to replicate these behavioral results in the communicative blocks.

Our neural hypotheses are driven by recent meta-analyses that distinguish between subfunctions in the mentalizing network [14,15]. One proposal is that the mPFC supports inferences about other people’s or your own lasting psychological and social states, such as personality traits, while the TPJ is involved in inferring temporary states of other people, such as immediate goals and intentions [15,16]. In this view, building an overall model of your interlocutor during a communicative task might rely especially on the mPFC, while faster, moment-by-moment adaptations to changes in common ground could involve the TPJ/pSTS [8]. In the present experiment, we therefore hypothesized that using a mental model of your addressee might especially engage the mPFC, because the information in these models mostly relates to enduring features of the addressee. The mPFC should therefore be sensitive to the general difference between communicative and non-communicative context. On the other hand, we expected that the TPJ should be especially engaged when speakers detect a relevant perspective difference during the communicative task blocks and need to adjust their language use accordingly. That is, we expected the TPJ to be sensitive to the distinction between information that is in common ground and information that is in privileged ground. Finally, given that speakers need to monitor the distinction between common and privileged ground in the communicative blocks, we expected that we might already find neural differences between the communicative and the non-communicative blocks during the viewing phase of the trials.

## Method

### Participants

Participants were recruited between May and December 2013. Twenty-four pairs of right-handed native Dutch speakers participated in the study. Participants did not know each other before the start of the experiment and signed up individually through the online university study participation system. All participants had normal or corrected-to-normal vision and no history of neurological disease. They gave written informed consent before the start of the experiment. Data from two pairs were excluded due to technical problems and data from two additional pairs were excluded due to excessive movement by the subject in the scanner (sudden movements of the head larger than 3.5mm in any direction). The results of the remaining forty participants (speakers: 22.55 years old, range 19–28 years old; 1 man; listeners: six men; 22.35 years old, range 18–28 years) are reported below. No participants dropped out during the study. Theory of mind localizer data from two participants were excluded from the analysis due to excessive motion during this task. The study was approved by the local ethics committee, Commissie Mensgebonden Onderzoek regio Arnhem-Nijmegen (CMO2001/095).

### Materials

**Fig 1:**
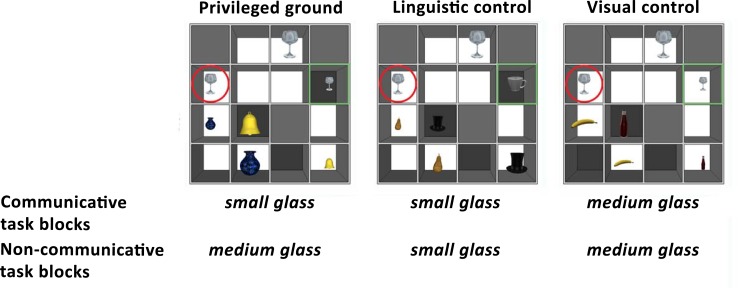
Overview of a triplet of trials from the speaker’s point of view, and the expected speaker responses in each of the six conditions. The task of the speaker was to describe a target object (red circle) for the listener (communicative blocks) or for him/herself (non-communicative blocks). In the communicative privileged ground condition, we expected speakers to take their addressee’s perspective into account (“small glass”). If they failed to take their addressee’s perspective into account, they could also describe the target object from their own perspective (“medium glass”). There was no relevant perspective difference in the other conditions. In the linguistic control conditions, speakers were expected to give the same verbal response as in the communicative priviliged ground condition. In the visual control conditions, both participants could see the competitor object that was occluded in the communicative privileged ground condition. Speakers thus saw the same number of relevant objects as in the privileged ground conditions. The green squares were added to the figure for clarification purposes to indicate the objects that differ between the privileged ground and the control conditions. They were not visible to the participants.

The materials were created in the same way as in Vanlangendonck et al. [10]. In that study, we distinguished between situations in which failing to take the addressee’s perspective into account either did (obligatory conditions) or did not (advisable conditions) threaten communicative success. We merged the obligatory and advisable conditions from this previous study in the current experiment. We manipulated the number, size and visibility of the relevant objects to create 6 conditions (Fig 1). In the privileged ground conditions (left column Fig 1), a competitor object was placed in a slot that was open only on the speaker’s side. In communicative trials, speakers had to ignore this competitor object in order to unambiguously describe the target object, because speakers knew that the listener could not see the competitor object. In non-communicative blocks, speakers did not have to ignore the additional competitor object, because there was no listener present (see the procedure section below). We created two types of control conditions, in which there was no occluded competitor object. In the linguistic control conditions (middle column Fig 1), the occluded object was replaced by another, unrelated object. As a result, speakers saw one relevant object fewer in these conditions than in the privileged ground conditions. This condition is called the linguistic control condition, since speakers were expected to produce the same verbal response in this condition as in communicative privileged ground trials in which they successfully adjusted their response based on their addressee’s perspective. In the visual control conditions (right column Fig 1), the object that was occluded in the privileged ground condition was visible to both participants. In these conditions, speakers therefore saw the same number of relevant objects as in the privileged ground conditions, hence we call these the “visual control” conditions.

Twelve different empty virtual arrays were used in the experiment. Each array was filled with six to eight objects chosen from a total of 22 objects. Objects were selected from the Object Databank, stimulus images courtesy of Michael J. Tarr, Center for the Neural Basis of Cognition and Department of Psychology, Carnegie Mellon University, http://www.tarrlab.org/. Each object could appear in four different sizes to make sure that participants could not rely on absolute size. Target/competitor objects and filler objects all appeared in sets of one, two or three objects of the same type to make sure that participants could not predict which objects would be relevant. The speaker and listener always saw the same total number of objects in a trial because we added filler objects to the occluded slots if needed. We created a unique stimulus list for each participant pair. Trials were presented in blocks of six trials that were created using the same array. The order of the trials within each block was randomized, and we randomized the blocks of trials so that neighboring blocks did not use the same array. More information about the trial creation and randomization can be found in Vanlangendonck, et al. [10].

### Procedure

Participants were randomly assigned to the roles of speaker and listener with a coin toss. The speaker performed the task in the MRI scanner, while the listener was seated in front of a computer in the MRI control room. The speakers spoke through a noise-cancelling microphone and listeners could hear the speaker over headphones. Participants completed four blocks of the main task (2 communicative and 2 non-communicative blocks), followed by a Stroop task localizer and a theory of mind localizer. The order of the four task blocks and the order of the localizers were counterbalanced. Each task block of the main experiment consisted of 60 trials, resulting in 240 trials in total. Before each block, speakers were informed whether the following block would be communicative or non-communicative.

**Fig 2:**
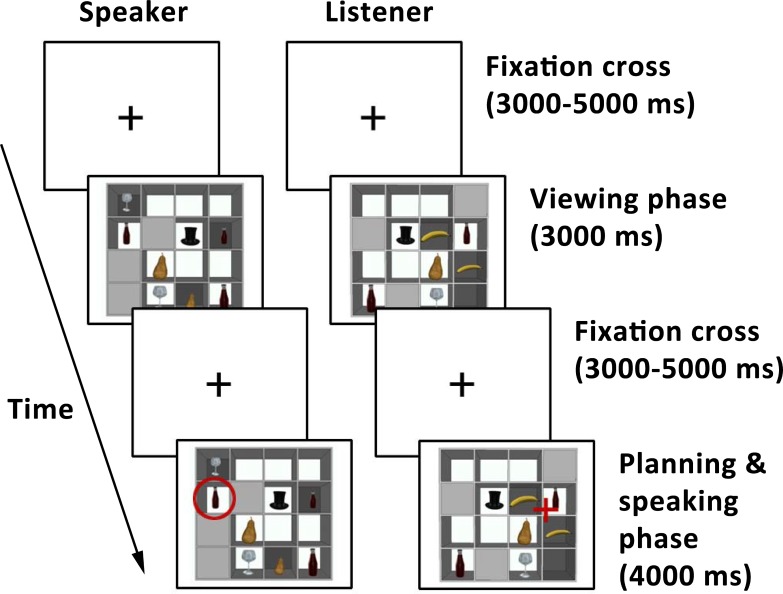
Trial sequence of a communicative trial from the speaker and the listener’s point of view. In the first phase of each trial, the speaker and the listener each viewed their side of the array. In the second phase of the trial, a red circle indicated which object the speaker had to describe. The speaker planned their response, pressed a button once they were ready to start speaking and then described the target object for the listener. At the same time, the listener tried to click on the intended object. The trial sequence in the non-communicative blocks looked identical, except that the listener did not take part in this task.

In communicative blocks, speakers and listeners played a referential communication game, in which the speaker described objects for the listener to select. Each trial featured a 4 x 4 array containing objects of different sizes. Each array contained three closed slots on each player’s side, allowing us to manipulate which objects were in common ground. Objects that were visible to both players were in (visual) common ground, while objects that were only visible to one player were in that player’s privileged ground (Fig 2). Each trial consisted of a viewing phase and a speaking phase. During the viewing phase, each player was shown his or her side of the array for 3000 ms. During the speaking phase (4000 ms), the speaker named the target object for the addressee (Fig 2). The target object was indicated using a red circle and was always in common ground. On the basis of the speaker’s description, the listener clicked on the intended object in his or her display. A variable jitter of 3000–5000 ms preceded each phase of the trial.

The listener did not participate in the non-communicative blocks of the task. In these blocks, the speaker saw the same type of arrays as in the communicative blocks, but they were told that the listener would not participate in these blocks. The speaker was therefore instructed to describe the cued objects for themselves. Based on this instruction, we expected speakers to produce responses that did not take the distinction between common and privileged information into account in these blocks (Fig 1).

Speakers were instructed not to use spatial descriptions, such as ‘leftmost’ or ‘third row’. Their verbal responses were recorded using a noise-cancelling microphone and they pressed a button when they were ready to start speaking, giving us a measure of the time they needed to plan their utterances. We recorded the location of the listeners’ mouse clicks, which allowed us to calculate the listeners’ accuracy. Before the start of the experiment, the speaker and the listener practiced the communicative task together using a real array and real objects. They then practiced the task together on a computer, and the speaker practiced the descriptive task by him/herself. The speaker also named all object pictures once before the start of the experiment to make sure they could easily recognize and name the objects.

After the experimental task, speakers completed a theory of mind localizer task [17–19]. We included this task to localize the mentalizing network. The false belief theory of mind localizer task shares an important feature with the communicative task, namely the requirement to represent different perspectives or beliefs. In both the communicative task and the localizer task, participants need to take into account what information is available to someone else and use this information to respond appropriately. During the localizer task, speakers were presented with twenty stories that required participants to represent false content. In half of the stories the false content concerned the physical state of an object (false photograph); in the other half of the stories it concerned another person’s belief (false belief). Each story was presented for ten seconds, after which participants were given a statement about the story to judge. They had to respond to the statements with a button press within five seconds. A variable inter-trial interval of 4000–8000 ms preceded each trial.

Participants also completed a Stroop localizer task based on Milham et al. [20]. A number of previous studies have shown that cognitive control plays a role in referential communication tasks (e.g., [21–24]), so we intended to use the main contrast of interest (incongruent > neutral) as a localizer for cognitive control processes. However, the incongruent > neutral contrast did not result in any significant clusters in our study and could therefore not be used for the planned analyses.

### Data acquisition and analysis

Participants were scanned in a Siemens 3T Skyra scanner using a 32-channel head coil. The functional images were acquired using an EPI multi-echo sequence (TR = 2250 ms; TE1 at 9 ms, TE2 at 19.3 ms, TE3 at 30 ms, TE4 at 40 ms; 36 slices; ascending slice order; slice thickness = 3 mm; slice gap = 0.3 mm; 64 x 64 matrix size; field of view = 212 x 212 mm; flip angle = 90°; voxel size = 3.3 x 3.3 x 3 mm). A high-resolution T1 image was acquired using an MPRAGE sequence (TR = 2300 ms; TE = 3.03 ms; 192 slices; voxel size = 1 x 1 x 1 mm, field of view = 256 × 256 × 192 mm).

Each of the four task blocks and the localizer tasks were scanned in separate runs. We acquired 30 additional functional scans before each block. These scans were used to calculate the optimal weighting of the five echoes, and this weighting matrix was applied to the remaining functional scans [25]. The functional images were processed using SPM8 (Statistical Parametric Mapping, www.fil.ion.ucl.uk/spm). The preprocessing of the functional images consisted of realignment to correct for head motion, slice timing correction to the onset of the middle slice, coregistration of the functional images to the T1 based on the subject-mean functional image, normalization to MNI space and spatial smoothing using a 3-dimensional isotropic Gaussian smoothing kernel (full-width half-maximum = 8 mm).

In the first-level statistical model, we included six event-types modeling the viewing phases (1 per condition), six event-types modeling the planning phases (1 per condition) and two event-types modeling the speaking phases (communicative and non-communicative). Events were modeled from picture onset until the button press for planning, and from the button press until picture offset for speaking. If the speaker had forgotten to press the button, we used a fixed planning duration of 900 ms. We also included six motion regressors per run. We ran an extra analysis with an additional regressor for the unexpected responses across conditions, but the results of this analysis were similar to the results of the reported analysis. A separate statistical model was created to analyze the theory of mind localizer. This model included four event-types (false belief stories, false photograph stories, false belief statements and false photograph statements) and six motion regressors. We used boxcar functions to model the durations (10 s for stories; 5 s for statements). All event-types from the main experiment and the localizer were convolved with the hemodynamic response function. Individual t-contrasts were created and used in second-level random-effect analyses. Group analyses were performed using one-sample t-tests. Whole-brain results were corrected for multiple comparisons by combining a p < 0.001 voxel-level threshold with a cluster extent threshold of 41 voxels. These settings were obtained by performing 2500 randomizations to assess which cluster extend level leads to false positive correction at a family-wise error rate of 5%. The combination of a voxel-level threshold with a cluster extent threshold is a good compromise between statistical sensitivity on the one hand and false positive error control on the other hand [26,27]. The script was retrieved from https://www2.bc.edu/sd-slotnick/scripts.htm.

For illustrative purposes, we computed parameter estimates for regions of interest (ROIs) in the mPFC, and left and right TPJ using Marsbar [28]. The ROIs were based on the mPFC and TPJ clusters found in the *communicative privileged ground > communicative linguistic control* and the *communicative privileged ground > communicative visual control* contrasts. We selected only voxels that were significantly activated in both contrasts. The temporoparietal clusters were part of a large, interconnected cluster, so we further limited these ROIs to voxels with x coordinates under -35 (left) or over 35 (right).

We coded the sound files for adjective use and we used speakers’ button press responses to determine the planning duration for each trial. We removed trials without a button press, trials without a response that could be coded and planning durations that were more than 3 standard deviations removed from the mean per subject from the dataset for the behavioral analysis (total 3.9% of trials removed). Listener performance was calculated by determining whether listeners clicked on the right slot within the 4000 ms response interval. We then analyzed speakers’ modifier use using logit mixed models and their planning durations using 2 x 3 repeated measures ANOVAs to investigate the effects of block (communicative or non-communicative), condition (privileged ground, linguistic control or visual control), and the interactions between these two factors. In addition, we tested whether we could replicate the results of our previous eye-tracking study [10] by comparing the communicative privileged ground condition to each of the communicative control conditions. All behavioral analyses were run in R version 3.0.3.

## Results

### Behavioral results

#### Manipulation checks

To check whether our block manipulation worked, we checked whether speakers indeed changed their strategy between the communicative and the non-communicative blocks. In the communicative blocks, we expected them to take their addressee’s perspective into account in the privileged ground trials (e.g., by saying “small glass” instead of “medium glass”). Indeed, we found that speakers produced mostly responses that took their addressee’s perspective into account in the communicative privileged ground condition (70.17%). Given that there was no addressee present in the non-communicative blocks, speakers did not have to adapt their responses to what someone else could see in the non-communicative privileged ground conditions. In line with this prediction, we found that speakers produced descriptions from their own perspective in 75.22% of trials in the non-communicative privileged ground condition. A paired t-test revealed that speakers produced significantly more responses that took their addressee’s perspective into account in the communicative privileged ground condition, t(19) = 4.63, p < 0.001. We hence conclude that the communicative manipulation was successful.

In the communicative blocks, listeners tried to select the object described by the speaker. Overall listener performance was high (83.47% correct), indicating that speakers and listeners understood the task.

#### Modifier use

**Fig 3:**
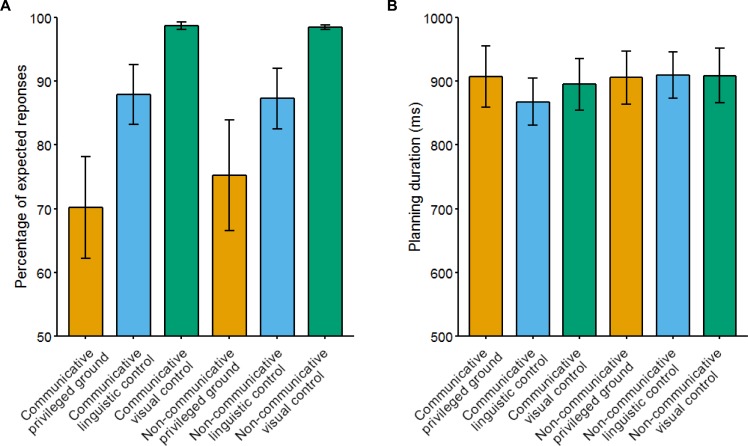
Percentage of expected responses and mean planning durations per condition. The expected responses were coded based on the predictions in Fig 1. Planning durations were calculated from picture onset until speakers pressed the button to indicate that they were ready to respond. Error bars indicate standard error of the mean.

Fig 3A shows the mean percentage of “expected responses”, i.e. responses that match the predictions in Fig 1. In the communicative privileged ground condition, we expected speakers to take their addressee’s perspective into account, while we did not expect speakers to take their addressee’s perspective into account in the non-communicative privileged ground condition. We analyzed the use of expected responses using logit mixed models [29]. We created a full model with random intercepts for subjects, and fixed effects of condition (privileged ground, linguistic control or visual control), block type (communicative or non-communicative) and the condition x block interaction. We tested the interaction and main effects through model comparison with reduced models omitting these effects. We found a significant main effects of condition (χ^2^(2) = 557.44, p < 0.001), but no significant main effect of block (χ^2^(2) = 0.60, p = 0.43) and no significant block x condition interaction effect (χ^2^(2) = 2.25, p = 0.33). Post-hoc paired t-tests revealed significantly more expected responses in the linguistic control conditions compared to the privileged ground conditions (t(19) = -2.97, p < 0.05) and in the visual control conditions compared to the linguistic control conditions (t(19) = -2.52, p < 0.05) after Holm-Bonferroni correction for multiple comparisons.

Speakers produced more expected responses in the communicative linguistic control condition compared to the communicative privileged ground condition, t(19) = 3.07, p < 0.01, and in the communicative visual control condition compared to the communicative privileged ground condition, t(19) = 3.68, p < 0.01. These findings are in line with previous studies with similar designs that showed that speakers generally take their addressee’s perspective into account in communicative tasks, although they cannot completely ignore privileged ground information [9–13]. The results of the linguistic control condition are below ceiling, because speakers sometimes produced overinformative responses by including a modifier when it was not necessary (see also the results of the advisable linguistic control condition in [10]).

#### Planning duration

An overview of the mean planning durations per condition can be found in Fig 3B. A 2 x 3 repeated-measures ANOVA with type of block (communicative or non-communicative) and condition (privileged ground, linguistic control or visual control) as factors did not reveal significant main or interaction effects (block: F(1,19) = 1.84, p = 0.19; condition: F(2,38) = 1.65, p = 0.21; block x condition: F(2,38) = 2.18, p = 0.13).

In order to compare these results with our previous eye-tracking study, we compared planning durations in the communicative privileged ground condition to each of the communicative control conditions using paired t-tests. We found that speakers took longer to plan their utterance in the communicative privileged ground condition compared to the linguistic control condition, t(19) = 2.53, p < 0.05. We did not find a significant difference in planning duration between the communicative privileged ground condition and the communicative visual control condition, t(19) = 0.82, p = 0.42.

### fMRI results

The goal of the present study was to investigate the neural mechanisms underlying speakers’ ability to take into account common ground during language production. First, we compared language planning and viewing in a communicative and a non-communicative context. We hypothesized that speakers would use a mental model of their addressee when planning an utterance in the communicative as compared to the non-communicative blocks. In addition, given that keeping track of the distinction between objects in common and in privileged ground only matters in the communicative blocks, we expected that speakers might use different strategies to explore the visual arrays before knowing which object to describe depending on whether they were in a communicative or a non-communicative context. Second, and most importantly, we compared language production in situations in which the speaker has to take into account common ground in order to communicate efficiently with their addressee and situations in which this is not necessary. This we explored in the comparison between communicative versus non-communicative privileged ground planning, as well as by comparing the communicative privileged ground planning condition to the two control conditions (linguistic, visual) in the communicative blocks. For the “viewing” analysis, we focused on the viewing phase of the trials (see Fig 2). For all other analyses, we focused on the planning phase, i.e. the time between the moment the speaker saw the array with a red circle around the target object and the moment they pressed the button to indicate that they were ready to start speaking.

#### Communicative vs. non-communicative privileged ground planning

**Fig 4:**
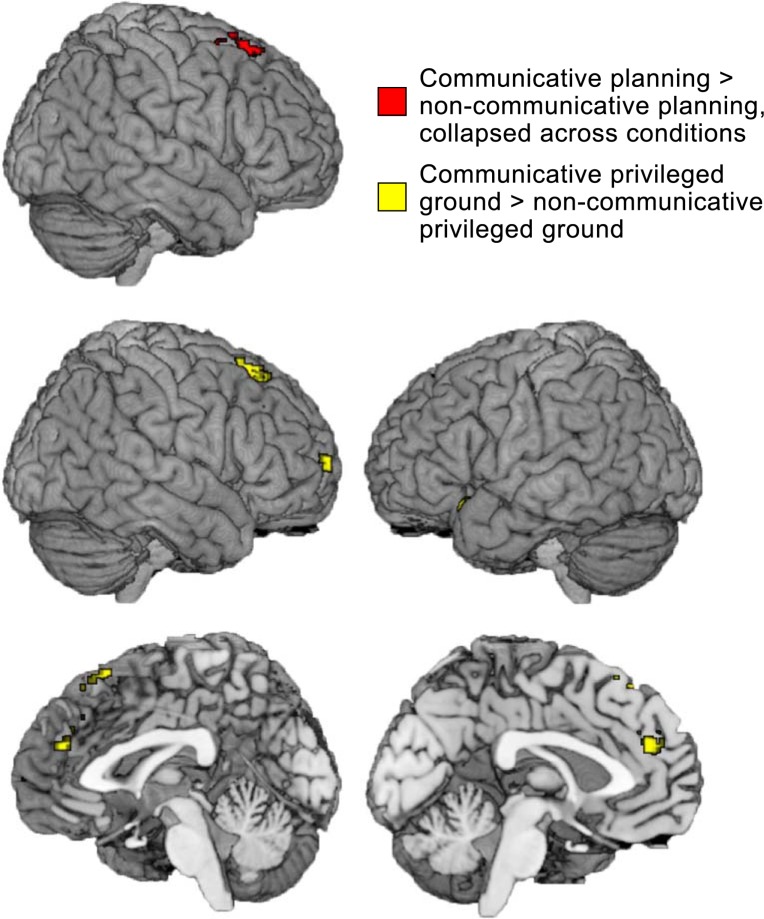
Brain areas showing greater activity in the communicative planning conditions compared to the non-communicative planning conditions (red) and the brain regions showing greater activity during the planning phase of the communicative privileged ground condition compared to the non-communicative privileged ground condition (yellow). In the latter comparison, the effect of communicative context was expected to be maximal.

We first compared the communicative and the non-communicative privileged ground trials because speakers only had to take their addressee’s perspective into account in the communicative privileged ground trials. The effect of communicative context may therefore be strongest when comparing these conditions directly. The contrast between planning in the communicative and non-communicative privileged ground conditions resulted in a series of clusters described in Table 1 and visualized in Fig 4. We found two right superior frontal clusters, one of which extends into the medial frontal gyrus, a left superior medial frontal cluster that extends into the left superior frontal gyrus, a cluster in the left insula and inferior frontal gyrus and a right inferior frontal cluster that extends into the right insula.

**Table 1:** Whole-brain results for task contrasts of interest.

Brain region	Cluster extent (voxels)	T value	MNI coordinates		
			x	y	z
**Communicative privileged ground planning > non-communicative privileged ground planning**					
right superior frontal gyrus	219	6.37	16	22	60
right superior frontal gyrus		5.19	18	30	54
right superior medial frontal gyrus		4.51	8	24	60
left superior medial frontal gyrus	199	5.04	-8	46	24
left superior frontal gyrus		4.66	-12	26	40
right anterior cingulate cortex		4.50	6	44	24
left insula	139	4.83	-34	20	-8
left inferior frontal gyrus (pars orbitalis)		4.72	-38	22	-16
left inferior frontal gyrus (pars orbitalis)		4.17	-40	32	-10
right superior frontal gyrus	41	4.40	22	66	6
right inferior frontal gyrus (pars orbitalis)	45	4.33	40	24	-10
right insula		4.24	32	20	-6
**Communicative privileged ground planning > communicative linguistic control planning**					
left inferior parietal lobule	6339	7.53	-48	-50	48
right superior occipital gyrus		7.50	36	-78	44
right superior parietal lobule		6.66	38	-58	60
left superior medial frontal gyrus	656	6.35	-8	30	42
right superior medial frontal gyrus		4.84	8	28	42
left supplementary motor area		4.38	-6	22	50
right middle frontal gyrus	619	5.92	48	30	34
right inferior frontal gyrus (pars opercularis)		5.10	50	20	38
right middle frontal gyrus		5.02	44	38	32
left middle frontal gyrus	394	4.93	-34	6	52
left middle frontal gyrus		4.71	-46	28	34
left middle frontal gyrus		4.67	-36	12	34
right insula	62	4.73	34	24	-6
right middle orbital gyrus	56	4.61	38	48	-8
right middle orbital gyrus		4.01	46	50	-8
right fusiform gyrus	162	4.55	38	-74	-18
right fusiform gyrus		3.95	42	-56	-16
right lingual gyrus		3.87	32	-82	-18
left insula	97	4.48	-30	22	0
left middle frontal gyrus	94	4.47	-44	50	8
left middle frontal gyrus		3.68	-38	46	4
left fusiform gyrus	82	4.36	-38	-72	-18
**Communicative privileged ground planning > communicative visual control planning**					
right angular gyrus	7803	7.15	54	-58	36
right superior parietal lobule		6.45	14	-64	58
right angular gyrus		6.43	36	-66	48
right middle frontal gyrus	3493	6.93	48	26	36
left superior middle gyrus		6.50	-4	32	38
right middle frontal gyrus		6.35	44	20	42
left middle frontal gyrus	1014	6.33	-38	12	36
left inferior frontal gyrus (pars triangularis)		5.03	-60	20	22
left middle frontal gyrus		5.01	-42	26	40
right superior frontal gyrus	653	6.12	34	62	14
right superior frontal gyrus		5.78	26	64	12
right middle frontal gyrus		4.93	36	64	2
right inferior frontal gyrus (pars orbitalis)	287	5.76	32	24	-8
right insula		4.68	32	28	2
right inferior frontal gyrus (pars orbitalis)		4.48	42	24	-16
right fusiform gyrus	133	4.68	40	-62	-20
right fusiform gyrus		4.05	34	-68	-16
right fusiform gyrus		3.73	26	-64	-12
right middle temporal gyrus	64	4.65	50	-42	-10
right middle temporal gyrus		4.30	58	-38	-10
right inferior temporal gyrus		3.88	58	-48	-10
left cerebellum	93	4.57	-36	-68	-22
left cerebellum		4.22	-30	-74	-22
left insula	65	4.33	-26	24	-4
**Communicative all conditions planning > non-communicative all conditions planning**					
right superior frontal gyrus	106	4.74	18	20	60
right superior frontal gyrus		4.30	20	6	64
right superior frontal gyrus		4.27	18	28	58
**Communicative all conditions viewing > non-communicative all conditions viewing**					
left middle occipital gyrus	241	5.75	-42	-80	6
left middle occipital gyrus		4.67	-36	-86	12
left superior occipital gyrus		4.29	-20	-96	18
right postcentral gyrus	362	5.60	42	-28	42
right inferior parietal lobule		4.35	44	-42	54
right precentral gyrus		4.30	38	-20	44
left calcarine gyrus	224	5.16	-14	-64	14
left calcarine gyrus		5.10	-12	-58	8
left middle cingulate cortex	61	5.03	-8	-34	50
right calcarine gyrus	205	4.89	6	-64	10
right calcarine gyrus		3.76	10	-56	12
right inferior temporal gyrus	66	4.51	48	-60	-6
right inferior occipital gyrus		4.00	40	-68	-6
right middle occipital gyrus	65	4.22	40	-78	22

#### Communicative privileged ground vs. linguistic and visual control planning

**Fig 5:**
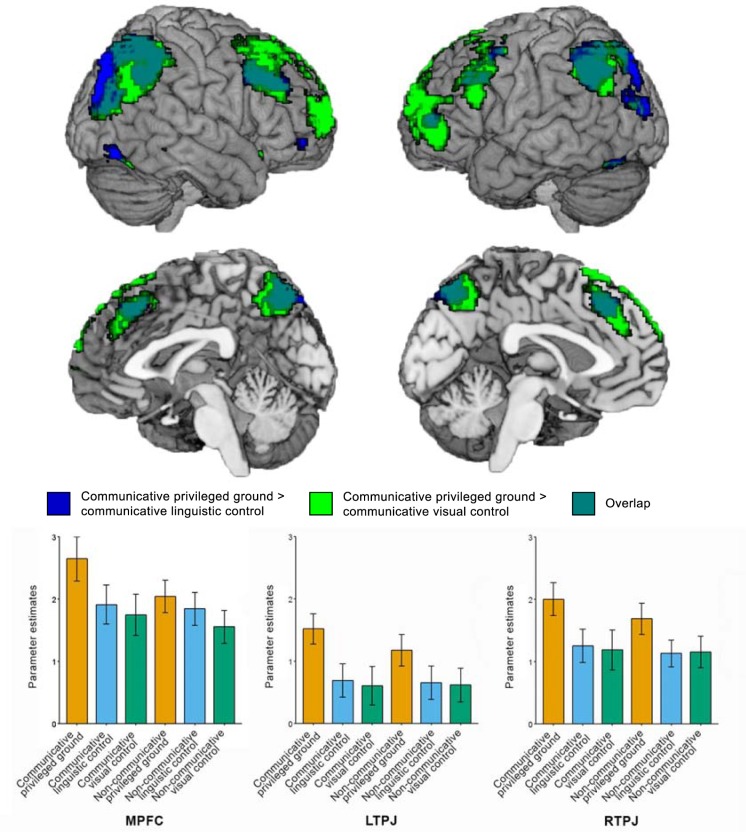
Brain areas showing greater activity during planning in the communicative privileged ground condition compared to the communicative linguistic control condition (blue) and the communicative visual control condition (green). The graphs below depict the parameter estimates in the different conditions in the mPFC, and the left and right TPJ. Error bars represent standard errors of the mean. Note that we did not test for statistically significant differences between the parameter estimates of different conditions in order to avoid making the non-independence error.

We compared planning in the communicative privileged ground condition to each of the control conditions. The results of these contrasts overlap considerably, as can be seen in Fig 5. The contrast between the communicative privileged ground condition and the communicative linguistic control condition resulted in a large cluster covering parts of the inferior and superior parietal lobule as well as the superior occipital gyrus. In the frontal lobe, we found a superior medial frontal cluster, a right orbitofrontal cluster and left and right middle frontal clusters. Finally, we found bilateral insula and fusiform activations. Similarly, the contrast between the communicative privileged ground condition and the communicative visual control condition resulted in a large bilateral cluster covering the superior parietal lobule and angular gyri. In addition, we found a number of left and right inferior frontal, middle frontal and superior frontal activations. We also found clusters in the right fusiform gyrus and the right middle temporal gyrus, as well as in the left insula and the left cerebellum.

#### Communicative vs. non-communicative planning

We compared speech planning in the communicative and the non-communicative blocks, collapsed over conditions. This resulted in one cluster in the right superior frontal gyrus (Fig 4, Table 1).

#### Communicative vs. non-communicative viewing

**Fig 6:**
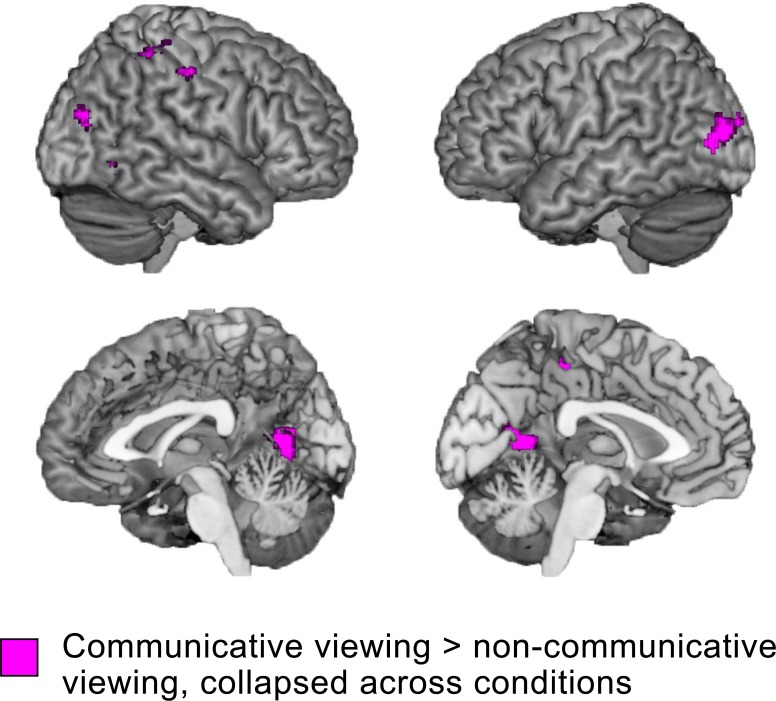
Brain areas showing greater activity in the communicative viewing conditions compared to the non-communicative viewing conditions.

Given that common ground is thought to be updated continuously during conversation [5], we expected that we may find neural differences even before speakers started to plan their utterances when comparing communicative and non-communicative task blocks. This comparison revealed clusters in the left and right middle occipital and calcarine gyri, as well as the right postcentral gyrus, the left cingulate cortex and the right inferior temporal gyrus (Fig 6, Table 1).

#### Theory of mind localizer: False belief vs. false photograph

**Fig 7:**
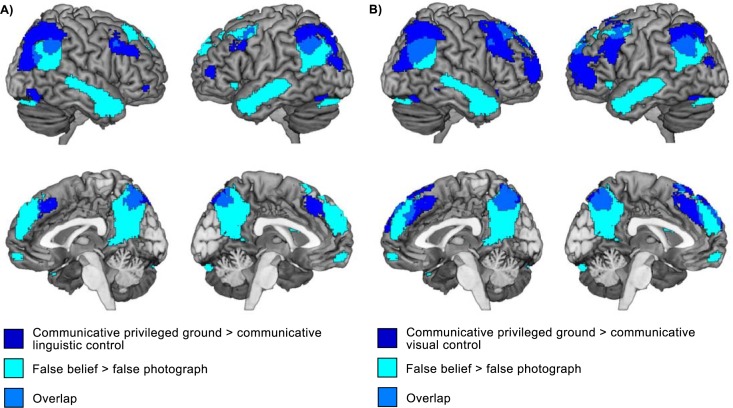
Brain areas showing greater activity during planning in the communicative privileged ground condition compared to the communicative linguistic control condition (A, blue), in the communicative privileged ground condition compared to the communicative visual control condition (B, blue) and in the false belief condition compared to the false photograph condition in the theory of mind localizer (A & B, cyan).

We compared the false belief and false photograph statements conditions from the theory of mind localizer. In this contrast, we found a large set of brain regions commonly found in theory of mind tasks including the bilateral TPJ, the precuneus and the mPFC (Table 2, Fig 7).

**Table 2:** Whole-brain results for comparison between false belief and false photograph statements.

Brain region	Cluster extent (voxels)	T value	MNI coordinates		
			x	y	z
**False belief > false photograph statements**					
left angular gyrus	1995	10	-56	-66	24
left middle temporal gyrus		7.77	-46	-56	22
left supramarginal gyrus		6.16	-60	-52	36
left precuneus	4297	9.7	2	-64	38
left precuneus		8.24	0	-56	40
left precuneus		7.53	-12	-50	40
right superior frontal gyrus	4999	8.69	16	46	34
right superior medial frontal gyrus		7.51	10	50	30
left superior frontal gyrus		7.2	-18	24	46
right angular gyrus	2143	8.38	48	-48	28
right angular gyrus		8.32	52	-62	26
right middle temporal gyrus		7.65	56	-60	18
left temporal pole	1453	7.23	-54	10	-32
left middle temporal gyrus		7.06	-62	-22	-12
left middle temporal gyrus		6.42	-54	-4	-20
right middle temporal gyrus	1565	6.95	54	4	-32
right temporal pole		6.59	50	20	-30
right middle temporal gyrus		6.56	58	-30	-2
right cerebellum	224	6.75	24	-80	-26
right cerebellum		4.5	44	-74	-24
left middle orbital gyrus	162	5.93	0	60	-12
left superior orbital gyrus		4.17	-14	58	-10
left middle orbital gyrus		3.81	-22	54	-10
left caudate nucleus	76	5.71	-14	8	20
left caudate nucleus		4.88	-12	-2	20
left cerebellum	395	5.67	-28	-76	-30
left cerebellum		5.67	-18	-88	-26
left cerebellum		4.87	-46	-72	-26
left inferior frontal gyrus (pars orbitalis)	46	4.81	-42	24	-8
left inferior frontal gyrus (pars orbitalis)		4.21	-48	28	-4

## Discussion

In this study, we examined the neural mechanisms that allow speakers to adapt their language use to moment-to-moment changes in common ground by manipulating which information they do or do not share with their addressee, in addition to comparing language production in a communicative and a non-communicative context. Behaviorally, we aimed to replicate the results of our previous study [10]. Neurally, we aimed to test under what conditions the mentalizing network contributes to communicative language production. We therefore mostly focus our discussion on brain regions that are part of this network.

### Behavioral results

Speakers generally took their addressee’s perspective into account when designing referring expressions in the communicative blocks, although they failed to ignore privileged information on all trials. These results are in line with previous findings using similar paradigms [9–13] and mostly results replicate the findings of our previous eye-tracking study [10]. Speakers produced slightly fewer expected responses in the communicative conditions in the current study compared to the previous study. One likely cause for this difference is that speakers switched between communicative and non-communicative blocks in the current experiment. Previous studies have shown that switching perspectives is costly once people have adopted a specific spatial perspective, even when switching to one’s own perspective [30,31]. Another difference is that in the current study, speakers’ planning durations were shorter in the communicative linguistic control condition than in the communicative privileged ground condition. Comparing the planning durations across conditions (Fig 3B) suggests that speakers were especially fast in this condition compared to the other conditions.

### fMRI results: Communicative privileged ground vs. non-communicative privileged ground planning

Speakers were only expected to adjust their language use based on their addressee’s perspective during speech planning in the communicative privileged ground condition. We therefore expected that the effect of communicative context on speech planning would be strongest in the direct comparison between speech planning in the communicative and the non-communicative privileged ground conditions. This contrast revealed clusters of activation in the right superior frontal gyrus, the left insula and inferior frontal gyrus, and in the dorsal mPFC.

The mPFC is a core region of the mentalizing network that supports the integration of social information over time [15]. It is thought to be subdivided into a more dorsal section, which is activated when thinking about the mental states of dissimilar others, and a ventral part, which is activated especially when thinking about the mental states of similar others [15,32]. Given that speakers and listeners did not know each other before the start of the experiment and that successful performance in the communicative privileged ground condition required speakers to focus on differences in perspective, it is unsurprising that the dorsal part of the mPFC was activated in this comparison. Our whole-brain results suggest that the mPFC is selectively engaged by the communicative privileged ground condition. The parameter estimates plotted in Fig 5 illustrate this. The mPFC thus appears to be especially activated when speakers perform a communicative task that requires them to adapt their language use to their addressee’s needs in order to communicate efficiently. These findings mimic the results of a comprehension study that found that adult listeners only show increased activation in the dorsal mPFC when they have to take a speaker’s perspective into account to respond appropriately [33].

### fMRI results: Communicative privileged ground vs. linguistic and visual control planning

When comparing the communicative privileged ground condition to each of the control conditions, we found a large, overlapping network of activations that include the core regions of the mentalizing network [14,15]: the mPFC and bilateral TPJ.

We found activations bilaterally in the dorsal/posterior part of the TPJ in this contrast. The TPJ has been proposed to be important for inferring temporary states of other people, such as goals, intentions, and desires, even when they differ from your own [15]. The posterior/dorsal part of this area may be especially important for the processing of mental perspectives [14]. Speakers’ visual perspective for relevant objects only differed from their addressee’s in the communicative privileged ground condition. The cluster we find in this area may therefore be the result of representing this perspective difference.

In addition to regions involved in mentalizing, we found bilateral clusters of activation in the ventro- and dorsolateral prefrontal cortex, which are known to play an important role in cognitive control processes (e.g., [34,35]). In the communicative privileged ground condition, speakers had to ignore an occluded competitor object. In contrast, all relevant objects were visible to both participants in the control conditions. Previous studies [21,23,24] have shown that inhibitory control skills correlate with people’s ability to take another person’s perspective into account during social interaction, suggesting that adapting your language use to another person’s perspective depends on your ability to inhibit your own perspective.

### fMRI results: Communicative vs. non-communicative planning

When comparing brain activity during speech planning in the communicative and non-communicative blocks, we found activation in a right superior frontal cluster. Although not considered one of the core components of the mentalizing network, it is interesting to note that this cluster appears in all contrasts we tested, as well in the theory of mind localizer. Similar right superior frontal activations have been found before in theory of mind tasks, including false belief tasks and tasks in which participants made trait judgments [14].

### fMRI results: Communicative vs. non-communicative viewing

The contrast between communicative and non-communicative viewing revealed a set of occipital and parietal clusters. One likely explanation for these findings is that speakers used a different strategy in the communicative and non-communicative viewing phases. The viewing phase allowed speakers to visually explore the objects in the array before seeing the cue indicating which object they had to describe. However, the distinction between objects in common and in privileged ground only mattered in the communicative blocks. Speakers may have therefore paid additional attention to the location of objects (in open or occluded slots) in the communicative blocks, resulting in increased activity in areas associated with visual attention [36]. Similar anticipation effects have been found in language comprehension [37].

### fMRI results: Theory of mind localizer

It is interesting to note that the clusters of activation we found in the comparisons between the conditions in the main task only partially overlap with the results of the theory of mind localizer. The temporoparietal clusters we found in the main task extend more dorsally compared to the cluster we found in the theory of mind localizer task, and we find little overlap between clusters in the mPFC. One possible explanation for these differences is that the theory of mind localizer we used here is not the most suitable task to tap into the perspective-taking processes speakers engaged in during the main task. Adapting your language use based on what your addressee can see requires relatively low-level visual perspective-taking (so-called level 1 perspective-taking). While both visual perspective-taking and false belief tasks require the representation of different perspectives, the neural correlates of these tasks do not completely overlap [38]. Alternatively, the limited overlap between the task contrasts and the localizer results may be due to statistical thresholding. In line with this possibility, we found increased overlap when we used a less stringent statistical threshold, especially in the TPJ.

### General discussion

In line with previous studies, our results show that adjusting your linguistic message for an addressee engages the mentalizing or theory of mind network. However, this study is the first to tease apart the effects of communicative context and the need to adjust your linguistic utterance to take common ground into account. Our results suggest that the mentalizing network plays a crucial role when speakers have to consider which information they share with their addressee in order to be informative. We extend previous findings by showing that the mPFC does not appear to be sensitive to communicative context per se, but rather becomes more activated when the communicative context has consequences for linguistic processing (i.e. when speakers have to take common ground into account to communicate efficiently). The TPJ, on the other hand, may be important for processing and representing your interlocutor’s perspective when it differs from your own. It may therefore be especially sensitive to potentially relevant perspective differences.

What do these findings mean for accounts of the neurobiology of language? One important finding is that the mentalizing network appears to be especially involved during communicative language processing when speakers have to take common ground into account. Our findings thus do not suggest that the mentalizing network always comes online when speakers design utterances for an addressee. Rather, areas involved in social cognition appear to be selectively activated when speakers need to take common ground into account in order to communicate efficiently. However, our results from the viewing phase show that being in a communicative as compared to a non-communicative context can lead to neural differences before language planning has started (i.e., before speakers knew which object they had to describe). While we did not collect eye-tracking data, a likely explanation is that speakers were more sensitive to the distinction between common and privileged information in the communicative viewing phases as compared to the non-communicative viewing phases. A recent MEG study [39] also reported neural differences between communicative and non-communicative settings before participants were presented with a communication problem. Combined, our results suggest that speakers monitored which objects were visible to their addressee during the viewing phase in the communicative blocks and then used this information to determine whether they had to adjust their linguistic utterance to take their addressee into account. This suggests that speakers in a communicative setting continuously update common ground and use this information to adapt their linguistic utterances based on their addressee’s needs. In the current experiment, speakers could relatively easily take common ground into account by considering which objects were visible to the other person. However, in real-life communicative settings, adapting your language use based on your addressee’s communicative needs can be more complex. For example, in order to communicative efficiently, speakers may also need to consider what their interlocutor knows and feels. In addition, it is important to note that during real-life conversations, the distinction between communicative situations in which speakers have to take common ground into account and situations in which this is not necessary is much less clear-cut than in the current study. Future research will have to clarify how the findings from the current study relate and generalize to communicative tasks that require more high-level perspective taking (e.g., considering what your interlocutor knows about a topic) and that incorporate more features of real-life social interactions (e.g., turn-taking).
